# Molecular mechanisms involved in HIV-1 Tat-mediated induction of IL-6 and IL-8 in astrocytes

**DOI:** 10.1186/s12974-014-0214-3

**Published:** 2014-12-24

**Authors:** Anantha Ram Nookala, Anil Kumar

**Affiliations:** Division of Pharmacology and Toxicology, UMKC-School of Pharmacy, 2464 Charlotte Street, Kansas City, MO 64108 USA

**Keywords:** HIV-1 Tat, Astrocytes, IL-6/IL-8, NF-κB, AP-1, PI3K/Akt, p38 MAPK, JNK MAPK

## Abstract

**Background:**

HIV-associated neurocognitive disorders (HAND) exist in approximately 50% of infected individuals even after the introduction of highly active antiretroviral therapy. HIV-1 Tat has been implicated in HIV-associated neurotoxicity mediated through production of pro-inflammatory cytokines like IL-6 and IL-8 by astrocytes among others as well as oxidative stress. However, the underlying mechanism(s) in the up-regulation of IL-6 and IL-8 are not clearly understood. The present study was designed to determine the mechanism(s) responsible for IL-6 and IL-8 up-regulation by HIV-1 Tat.

**Methods:**

SVG astrocytes were transiently transfected with a plasmid encoding HIV-1 Tat. The HIV-1 Tat-mediated mRNA and protein expression levels of both IL-6 and IL-8 in SVG astrocytes were quantified using real time RT-PCR and multiplex cytokine assay respectively. We also employed immunocytochemistry for staining of IL-6 and IL-8. The underlying signaling mechanism(s) were identified using pharmacological inhibitors and siRNA for different intermediate steps involved in PI3K/Akt, p38 MAPK and JNK MAPK pathways. Appropriate controls were used in the experiments and the effect of pharmacological antagonists and siRNA were observed on both mRNA expression and protein levels.

**Results:**

Both IL-6/IL-8 mRNA and protein showed peak expressions at 6 hours and 96 hours post-transfection, respectively. Elevated levels of IL-6/IL-8 were also confirmed by immunocytochemistry. Our studies indicated that both NF-kB and AP-1 transcription factors were involved in IL-6 and IL-8 expression mediated by HIV-1 Tat; however, AP-1 was differentially activated for either cytokine. In the case of IL-6, p38δ activated AP-1 whereas JNK but not p38 MAPK was involved in AP-1 activation for IL-8 production. On the other hand both PI3K/Akt and p38 MAPK (β subunit) were found to be involved in activation of NF-κB that led to IL-6 and IL-8 production.

**Conclusion:**

Our results demonstrate HIV-1 Tat-mediated induction of both IL-6 and IL-8 in a time-dependent manner in SVG astrocytes. Furthermore, we also showed the involvement of NF-κB and AP-1 transcription factors regulated by PI3/Akt, p38 MAPK and JNK MAPK upstream signaling molecules. These results present new therapeutic targets that could be used in management of HAND.

**Electronic supplementary material:**

The online version of this article (doi:10.1186/s12974-014-0214-3) contains supplementary material, which is available to authorized users.

## Background

One of the hallmarks of neurodegeneration is inflammation in the central nervous system and dysregulation of cytokines and chemokines has been attributed to this process. Several pro-inflammatory cytokines, including IL-1β, IL-6, IL-8 and TNF-α have been implicated in neuroinflammation in a variety of neurodegenerative diseases including Alzheimer’s disease [1], Parkinson’s disease (PD) [2], multiple sclerosis [3] and HIV-associated neurocognitive disorders (HAND) [4]. In particular, elevated levels of cytokines have been reported to correlate with the degree of HAND [5]. While IL-6 and IL-8 have been extensively studied for their role in Alzheimer’s disease and PD, not much is known about the role of these cytokines in HAND.

Introduction of combined antiretroviral therapy has significantly reduced the incidence of HIV-associated dementia (HAD), but increased the prevalence of less severe forms of cognitive dysfunctions [6]. Many agents have been implicated in leading to HAND, including whole virus as well as viral proteins. The role of two HIV proteins, HIV-1 Tat (trans activator of transcription) and gp120 has been extensively studied for their role in neuroinflammation. We and others have recently shown that gp120 induces pro-inflammatory cytokines and reactive oxygen species in different cells of the brain and thereby contributes to HAND [7,8]. Likewise, HIV-1 Tat has also been shown to promote the release of several pro-inflammatory cytokines and reactive oxygen species from different brain cells [9,10].

HIV-1 Tat is an accessory viral protein produced at a very early stage of HIV-1 replication and increases the transcription of HIV-1 genome by greater than 100 fold [11]. In addition to its role in replication, HIV-1 Tat has also been shown to be involved in central nervous system damage by various mechanisms, including the apoptosis of the neurons by increasing the intracellular calcium concentration and activation of the N-methyl-D-aspartate receptors [12]. It also affects the function of dopamine neurotransmission by deregulating the functions of dopamine transporter and vesicular monoamine transporter [13]. In addition to its direct effect on neurons, HIV-1 Tat has been shown to exhibit a bystander effect on neurons by promoting the release of several pro-inflammatory cytokines/chemokines from astrocytes and microglia [14,15]. HIV-1 Tat also affects the integrity of the blood brain barrier by disrupting the tight junction proteins in brain microvascular endothelial cells [16].

Astrocytes comprise the majority of cells in the brain and also represent an important reservoir for the production of various mediators of inflammation, particularly in response to HIV-1 [17,18]. They play important roles in many brain functions, including the modulation of neuronal activity and regulation of synaptic plasticity [19,20]. *Post-mortem* studies of brain samples of HIV-1-infected patients showed that a small portion of astrocytes are restrictively infected with the virus [21]. In a previous study, Churchill and colleagues have demonstrated extensive astrocyte infection by HIV-1 in individuals suffering from HAND [22]. In yet another independent study, it has been shown that a sub-population of latently infected astrocytes undergo apoptosis that correlates with the extent of HAND [18]. HIV-1 Tat has not only been shown to be produced by the HIV-1-infected astrocytes [23], but has also been shown to promote the up-regulation of a variety of cytokines/chemokines including MCP-1 (monocyte chemotactic protein 1), IL-8, IL-6 and TNF-α [10,24,25].

Although HIV-1 Tat has been shown to induce IL-6 and IL-8 in astrocytes, the mechanism(s) remains largely unknown. The present study was undertaken to ascertain underlying mechanism(s) for IL-6 and IL-8 cytokine expressions with the idea of identifying transcription factors and upstream signaling molecules.

## Materials and methods

### Cell culture and reagents

Experiments were performed using SVG astrocytes, originally developed by Dr. Eugene Major and colleagues and primary astrocytes (obtained from BDRL, Seattle, WA, USA). The cells were cultured in DMEM supplemented with sodium bicarbonate, non-essential amino acids, L-glutamine, fetal bovine serum and gentamicin and were maintained in an incubator at 37°C and humidified air with 5% CO_2_. HIV-1 Tat expression plasmid, initially developed by Dr. E Verdin, Gladstone institute, UCSF (catalog # 10453), and HIV-1 Tat protein (catalog # 2222) were obtained from the NIH AIDS reagent program. All the pharmacological inhibitors were purchased from Cayman Chemical Company (Ann Arbor, MI, USA). siRNA against p38 isoforms (α/β/γ/δ), p50, p65 and negative silencer1 (scrambled) were purchased from Ambion Inc. (Carlsbad, CA, USA). siRNA against Akt isoforms (1/2/3), AP-1 (c-jun), C/EBPα and C/EBPγ were procured from Thermo Fisher Scientific (Pittsburgh, PA, USA). The primary antibodies for p65, p-c-jun, glyceraldehyde 3-phosphate dehydrogenase (GAPDH) and all the secondary antibodies were purchased from Cell Signaling (Danvers, MA, USA) and primary antibodies for p-p38, p-Akt, p-JNK and LaminB were purchased from Santa Cruz Biotechnology (Dallas, TX, USA).

### Transfection

SVG astrocytes were transiently transfected with HIV-1 Tat plasmid by using Lipofectamine 2000 (Life Technologies, NY, USA) as previously described [26]. Briefly, astrocytes were plated in a 6- or 12-well plate and allowed to adhere overnight. The complete DMEM was removed the following day and the cells were washed twice with PBS and serum free DMEM was added to the wells. A transfection mixture containing Lipofectamine and Optimem with or without HIV-1 Tat plasmid was added to the wells. After 5 hours, the transfection mixture was replaced with complete DMEM. For experiments involving pharmacological inhibitors, cells were pretreated 1 hour prior to the transfection. For experiments involving siRNA, the cells were transfected with siRNA for 48 hours before transfection with HIV-1 Tat plasmid to ensure optimum knock down.

### Real time RT-PCR and multiplex cytokine assay

RNA was extracted from the SVG astrocytes using RNeasy kit from Qiagen (Valencia, CA, USA) as recommended by the manufacturer. Primer sequences and PCR conditions for IL-6 and IL-8 have been published previously [7,27]. The expression levels of these cytokines were normalized with hypoxanthine phosphoribosyl transferase. The fold expression of cytokines was calculated using the 2^-ΔΔct^ method.

The protein concentrations of IL-6 and IL-8 in the culture supernatants were determined by multiplex cytokine assay. The detailed protocol has been published previously [26]. Briefly, 50 μL of the supernatants or standards are added to a pre designed 96-well plate containing magnetic beads. After incubation for 30 minutes at room temperature, 25 μL of detection antibody are added to each well. Then wells were washed and 50 μL of streptavidin-PE conjugate is added and incubated for 10 minutes, followed by washing and adding assay buffer. The concentrations of IL-6 and IL-8 were determined and analyzed by Bio-Plex manager 5 software using 5-PL statistics (Bio-Rad, Hercules, CA, USA).

### Western blot

SVG astrocytes were harvested and whole cell lysates as well as cytoplasmic and nuclear extracts were prepared as needed. For whole cell extract preparation, radioimmunoprecipitation assay (RIPA) buffer was used to lyse the cells, followed by homogenization and spinning at 10,000 rpm for 10 minutes to remove the cell debris. For nuclear and cytoplasmic extracts, the cells were trypsinized and centrifuged at 10,000 rpm for 5 minutes. The cell pellet was resuspended in 300 μL of cytoplasmic extraction reagent, followed by incubation on ice and washing with PBS. Then 200 μL of nuclear extraction reagent were added and the cell pellet was incubated on ice for 15 minutes to collect the nuclear portion. The concentrations of the proteins were determined from the standard curve using the BCA kit (Pierce Biotechnology, Rockford, IL, USA). Twenty micrograms of protein sample were loaded into the wells of 12% polyacrylamide gel and the sample was electrophoresed at 80 V for 3 hours followed by transfer onto a polyvinylidene fluoride (PVDF) membrane for 90 minutes at 350 mA. The membrane was blocked in 5% nonfat dry milk for 1 hour. The membranes were incubated in primary antibody overnight at 4°C. The membranes were washed with PBST and incubated in the appropriate horseradish peroxidase (HRP)-conjugated secondary antibody for 90 minutes. The membranes were again washed with PBST and the proteins were visualized by using BM Chemiluminescence Western Blotting Substrate (POD) (Roche Applied Sciences, Indianapolis, IN, USA). The bands were analyzed and quantified by Fluorchem HD2 software (Alpha Innotech, San Leandro, CA, USA). GAPDH and LaminB were used as internal loading controls for cytoplasmic and nuclear protein, respectively, to normalize the expression of proteins of interest.

### Immunocytochemistry

SVG astrocytes were plated in a 12-well plate with cover slips and were allowed to adhere overnight. Next morning, complete DMEM was replaced by DMEM without serum and cells were transfected with the plasmid encoding HIV-1 Tat for 24 hours. Golgi stop (1 mg/ml) was added 6 hours before the termination of transfection to prevent the release of IL-6 and IL-8 from astrocytes. The cells were fixed in 1:1 solution of ice-cold acetone and methanol for 20 minutes and were allowed to air dry for 5 minutes. The cells were washed with PBST and blocked with 1% BSA in PBST for 30 minutes. Then the cells were incubated overnight with a mixture of primary antibodies, including mouse anti-glial fibrillary acidic protein (anti-GFAP) (1:1,000) and either rabbit anti-IL-6 or rabbit anti-IL-8 (1:500). The cells were washed thrice with PBST and incubated with a mixture of secondary antibodies containing Alexafluor 488 labeled anti-rabbit IgG and Alexafluor 555 labeled anti-mouse IgG at a dilution of 1:1,000. After incubating the cells in the dark for 1 hour, the cells were washed thrice with PBST and the cover slips were mounted on a glass slide containing 4′,6-diamidino-2-phenylindole (DAPI) (Vector Laboratories, Burlingame, CA, USA). The images were acquired using inverted confocal microscope, Leica TCS SP5 II (Leica Microsystems, Wetzler, Germany). ImageJ software was used to analyze the images and calculate the intensity values by using GFAP as housekeeping gene.

### Statistical analysis

The statistical significance for the time kinetics experiments was calculated using Student’s *t*-test. For all the experiments involving the use of pharmacological antagonists and siRNA, one-way analysis of variance (ANOVA) was used to calculate the statistical significance. All the experiments were performed in triplicates and the results are represented by the mean ± standard error (SE) of at least three independent experiments. A *P*-value of ≤ 0.05 was considered to be statistically significant.

## Results

### HIV-1 Tat induces a time-dependent expression of IL-6 and IL-8 in astrocytes

**Figure 1:**
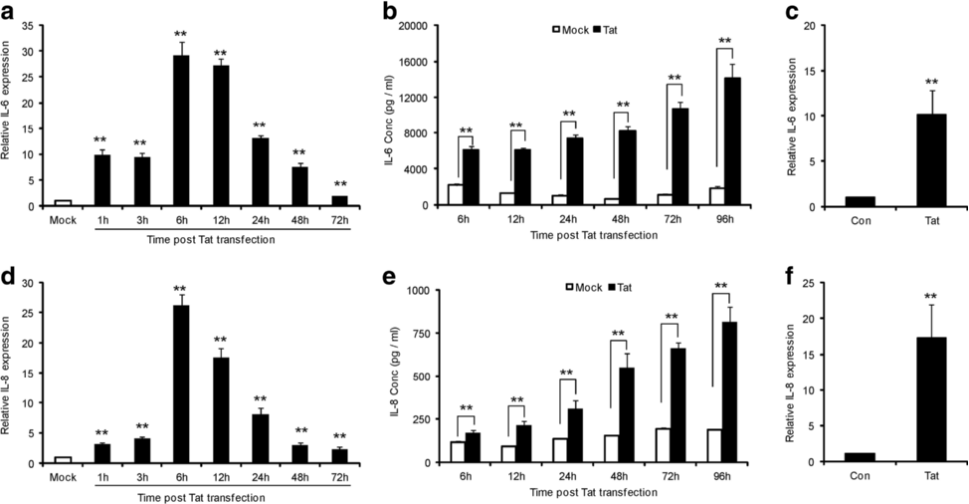
**HIV-1 Tat induces time dependent expression of IL-6 and IL-8 in SVG astrocytes.** Seven hundred thousand SVG astrocytes were transfected with 0.3 μg of HIV-1 Tat plasmid using Lipofectamine 2000. **(a, d)** Cells were harvested at 1, 3, 6, 12, 24, 48 and 72 hours and total RNA was isolated. The expression levels of IL-6 and IL-8 were determined by real time RT-PCR. The values represented are normalized to their mock-transfected controls. **(b, e)** The concentrations of IL-6 and IL-8 in cell culture supernatants were measured at 6, 12, 24, 48, 72 and 96 hours after transfection by multiplex cytokine assay. Open bars and closed bars represent protein concentrations of mock- and HIV-1 Tat- transfected cells, respectively. Each experiment was done at least in triplicate and each bar represents the ± SE of three individual experiments. **(c, f)** Primary astrocytes from 2 independent donors were treated with 200 ng/ml of Tat protein for 2 hours and total RNA was isolated. The expression levels of IL-6 and IL-8 were determined by real time RT-PCR. Statistical analyses was performed by Student’s *t*-test and ** denotes *P*-value of ≤ 0.01.

In the present study, we first sought to confirm the earlier finding that HIV-1 Tat up-regulates IL-6 and IL-8 expression in astrocytes. The SVG astrocytes were transfected with a plasmid encoding HIV-1 Tat and the transfection efficiency was monitored by setting a parallel transfection with a plasmid encoding green florescent protein [27]. The efficiency as measured and analyzed by flow cytometry ranged between 50 to 65% (data not shown). We first performed a time kinetics experiment to determine the peak expression levels of IL-6 and IL-8 at mRNA and protein levels. Compared to mock-transfected cells, the levels of IL-6 and IL-8 mRNA increased as early as 1 hour, peaked at 6 hours and declined steadily until 72 hours (Figure 1a, d). The peak expression levels of IL-6 and IL-8 after 6 hours of transfection was found to be 29.2 ± 2.5 and 26.2 ± 1.7 fold, respectively.

To determine the protein levels of these cytokines, a multiplex cytokine assay was performed at indicated time points (6, 12, 24, 48, 72 and 96 hours). The levels of IL-6 protein started to increase significantly from 6 hours (6.10 ± 0.36 ng/ml versus 2.10 ± 0.19 ng/ml) after transfection and gradually increased until 96 hours (14.08 ± 1.59 ng/ml versus 1.79 ± 0.22 ng/ml) where it showed the maximum increase (until observation period) compared to the mock-transfected cells (Figure 1b). Similar to IL-6, the protein expression of IL-8 has also started to increase significantly from 6 hours (0.172 ± 0.01 compared to 0.112 ± 0.006 ng/ml in controls) and showed a progressive increase in protein expression until 96 hours (0.813 ± 0.09 ng/ml compared to 0.185 ± 0.005 ng/ml in controls) (Figure 1e). There was no significant change in IL-6 and IL-8 protein levels in mock-transfected cells. These results clearly demonstrate that HIV-1 Tat induces the expression of IL-6 and IL-8 from astrocytes in a time-dependent manner at mRNA as well as protein level. These results were also confirmed in primary astrocytes isolated from 2 fetal brains collected after abortion where treatment with HIV-1 Tat protein (200 ng/ml) for 2 hours showed significant IL-6 and IL-8 up-regulation (Figure 1c, f).

**Figure 2:**
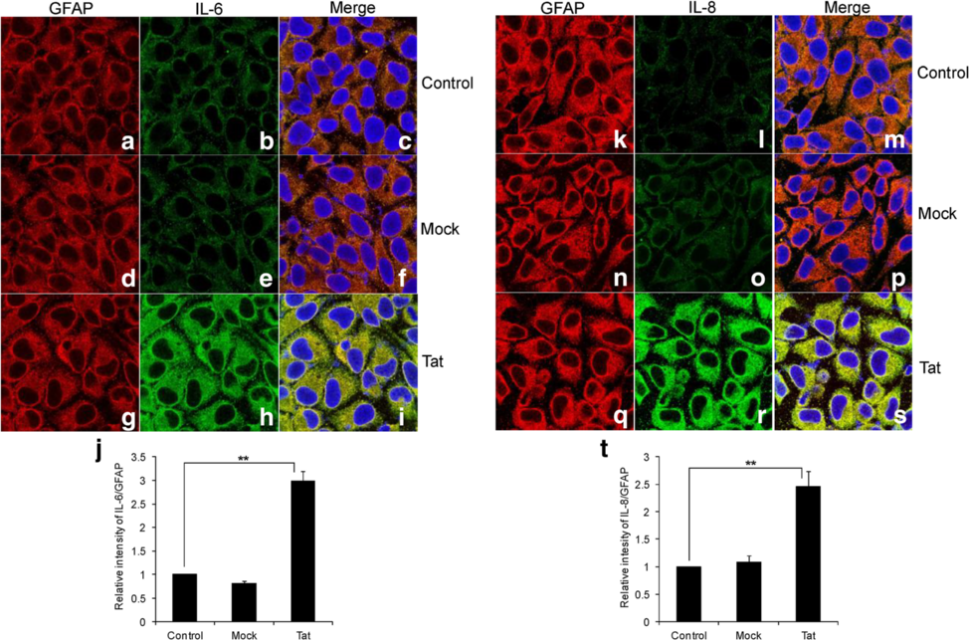
**Immunocytochemistry of IL-6 and IL-8 mediated by HIV-1 Tat in astrocytes. (a-i, k-s)** Five hundred thousand SVG astrocytes were grown on a cover slip and transfected with plasmid encoding HIV-1 Tat. The over expression of IL-6 and IL-8 by HIV-1 Tat **(g-i, q-s)** was compared with control cells **(a-c, k-m)** and mock-transfected cells **(d-f, n-p)**. The cells were costained with a mixture of antibodies against GFAP (red) and either IL-6 or IL-8 (green). The nucleus was stained blue using 4′,6-diamidino-2-phenylindole (DAPI). The images for different fluorophores were obtained using an inverted confocal microscope. The quantification of IL-6 and IL-8 were done using imageJ software **(j, t)**. Each experiment was done at least in triplicate and each bar represents the ± SE of three individual experiments. Statistical analyses was performed by Student’s *t*-test and ** denotes *P*-value of ≤ 0.01.

Immunocytochemistry was performed to determine the presence of elevated levels of IL-6 and IL-8 proteins inside the cell when they are transfected with HIV-1 Tat plasmid (Figure 2). The results clearly show that both IL-6 and IL-8 are increased in HIV-1 Tat-transfected cells when compared with mock-transfected and untransfected cells (Figure 2h, 2r). Our results also indicate that astrocyte marker GFAP did not significantly change with either control or mock or HIV-1 Tat transfection (Figure 2a, d, g, k, n, q). The intensity of IL-6 over GFAP was 3 ± 0.21 fold higher in HIV-1 Tat-transfected cells when compared to the control cells (Figure 2j). Similarly, the intensity of IL-8 was 2.46 ± 0.28 fold more when compared to the untransfected cells (Figure 2t). The change in intensities of either IL-6 or IL-8 was not significant in mock-transfected cells when compared to the untransfected cells.

### HIV-1 Tat-mediated over-expression of IL-6 and IL-8 involves NF-κB pathway

**Figure 3:**
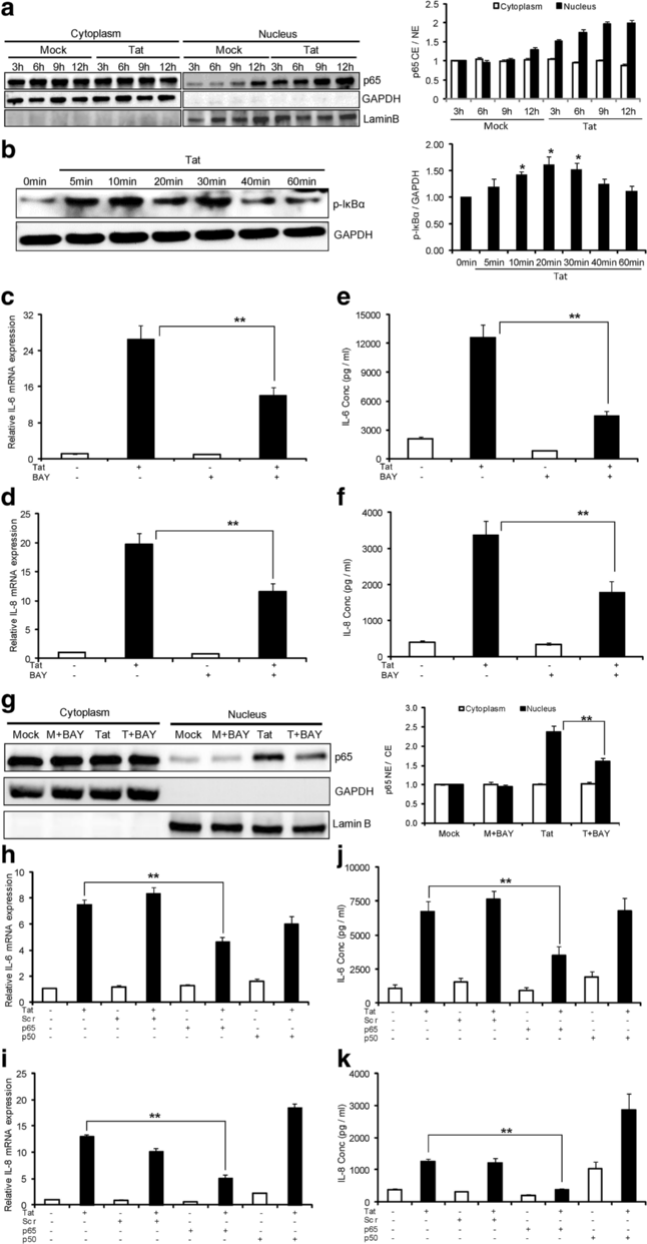
**Involvement of NF-κB in HIV-Tat mediated up-regulation of IL-6 and IL-8. (a)** SVG astrocytes were either mock-transfected or transfected with HIV-1 Tat plasmid and translocation of p65 was measured at 3, 6, 9 and 12 hours. Open bars and closed bars represent cytoplasm and nuclear fractions, respectively. **(b)** Primary astrocytes were treated with 200 ng/ml Tat protein and p-IκBα protein levels were measured from 0 minutes to 60 minutes. The bar graph represents the mean values obtained from two independent donors. **(c-g)** Astrocytes were pretreated with 10 μM concentration of NF-κB inhibitor (BAY11-7082) 1 hour prior to the transfection. The expressions of IL-6 and IL-8 were determined at 6 hours and 48 hours post transfection for mRNA **(c, d)** and protein **(e, f)**, respectively. The values represented are normalized their mock-transfected controls. **(g)** Astrocytes were either mock-transfected or transfected with HIV-1 Tat plasmid for a duration of 6 hours and translocation of p65 was measured. **(h-k)** Astrocytes were transfected with siRNA (scrambled or p65 or p50) for 48 hours, followed by transfection with HIV-1 Tat plasmid. The expression of IL-6 and IL-8 was determined at 6 hours and 48 hours post transfection for mRNA **(h, i)** and protein **(j, k)**, respectively. Each experiment was done at least in triplicate and each bar represents the ± SE of three individual experiments. Statistical analyses was performed by one-way ANOVA and ** denotes *P*-value of ≤ 0.01.

NF-κB is a crucial transcription factor involved in many inflammatory processes, including the up-regulation of several pro-inflammatory cytokines. Previous reports from our group and others have demonstrated the role of NF-κB in the production of cytokines by HIV-1 Tat in astrocytes and other cells [26,28]. We performed time kinetics to determine the peak increase in p65 translocation (indicator of NF-κB activation). Compared to mock-transfected cells, p65 translocation in HIV-1 Tat-transfected cells started to increase as early as 3 hours, peaked at 9 hours and remained constant until 12 hours (Figure 3a). We also confirmed the involvement of NF-κB in primary astrocytes from two different donors by measuring p-IκBα expression after treatment with HIV-1 Tat protein. The level of p-IκBα increased as early as 5 minutes and peak level was observed at 20 minutes post-treatment (Figure 3b).

After establishing that HIV-1 Tat-induced p65 translocation with HIV-1 Tat in SVG astrocytes, we wanted to determine if NF-κB was involved in up-regulation of IL-6 and IL-8 cytokines. For this, we first used a pharmacological inhibitor. Cells were pretreated with 10 μM concentration of specific inhibitory kinase kinase (IKK) inhibitor, BAY11-7082 1 hour prior to transfection with HIV-1 Tat. The levels of IL-6 and IL-8 were determined at 6 hours and 48 hours post transfection for mRNA and protein respectively. BAY11-7082 decreased the expression of IL-6 by 47.1 ± 6.1% and 63.3 ± 4.7% at mRNA and protein levels respectively (Figure 3c, e). Similarly, the expression levels of IL-8 were decreased by 41.1 ± 7.5% and 47.4 ± 4.7% at the levels of mRNA and protein respectively (Figure 3d, f). We also measured the translocation of p65 into the nucleus upon pretreatment with BAY11-7082. The translocation of p65 decreased by 32 ± 3.3% compared to the HIV-1 Tat-transfected cells (Figure 3g). To confirm the results of the pharmacological inhibitor, we employed a siRNA approach to individually knock down major subunits of NF-κB which include p50 (NF-κB1) and p65 (RelA) subunits. We have previously verified the efficiency of knock down of individual siRNA [29]. Our results show that p65, but not p50 knock down decreased the expression of IL-6 by 40.8 ± 3.8% and 48.2 ± 5.8% at mRNA and protein levels respectively (Figure 3h, j). Similar to the inhibitor results, knock down of p65 decreased the expression levels of IL-8 by 60.1 ± 4.7% at the level of mRNA and by 69.3 ± 2.6% at the level of protein (Figure 3i, k). Knock down of the p50 subunit of NF-κB did not affect the expression levels of IL-6 and IL-8 at either level of mRNA or protein. Knock down of p50 subunit of NF-κB did not have any effect on the expression levels of IL-6, but has increased the expression levels of IL-8 at mRNA and protein.

### HIV-1 Tat-mediated induction of IL-6 and IL-8 involves p38 MAPK pathway

**Figure 4:**
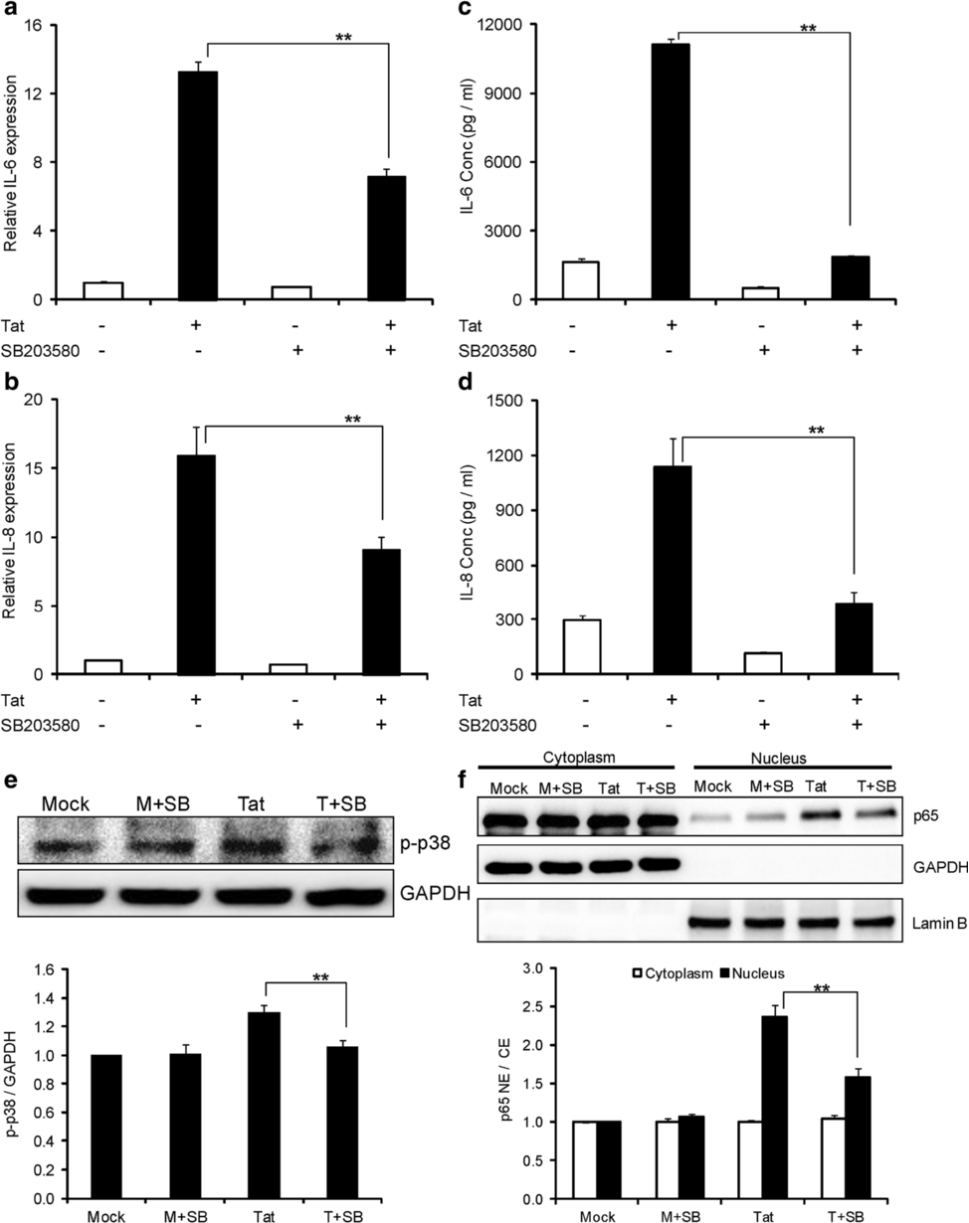
**Inhibition of HIV-1 Tat-induced expression of IL-6 and IL-8 by inhibitors of p38 mitogen activated-protein kinase (MAPK). (a-f)** Astrocytes were pretreated with 10 μM concentration of p38 MAPK inhibitor (SB203580) 1 hour prior to the transfection. **(a, b)** The expression levels of IL-6 and IL-8 at mRNA were determined by real time RT-PCR at 6 hours post transfection. The values represented are normalized to their mock-transfected controls. **(c, d)** The protein concentrations of IL-6 and IL-8 were determined in the cell culture supernatants at 48 hours post transfection by multiplex cytokine assay. **(e)** Astrocytes were either mock-transfected or transfected with HIV-1 Tat plasmid for a duration of 6 hours and p-p38 levels were measured in whole cell extracts. **(f)** Astrocytes were either mock-transfected or transfected with HIV-1 Tat plasmid for duration of 6 hours and translocation of p65 was measured. A representative Western blot is shown in panels **(e)** and **(f)**. Each experiment was done at least in triplicate and each bar represents the ± SE of three individual experiments. Statistical analyses was performed by one-way ANOVA and ** denotes *P*-value of ≤ 0.01.

After establishing the role of NF-κB, we wanted to explore the role of upstream signaling molecules that could activate NF-κB. p38 mitogen activated-protein kinase (p38 MAPK) is a family of important upstream MAPKs that can activate NF-κB and regulate the expression of many cytokine/chemokines. To determine the role of p38 MAPK in the up-regulation of IL-6 and IL-8 by HIV-1 Tat, we used SB203580, a specific inhibitor of p38 MAPK. Pretreatment of astrocytes with 10 μM concentration of SB203580 decreased the expression levels of IL-6 by 47.8 ± 4.1% and 82.4 ± 0.5% at levels of mRNA and protein respectively (Figure 4a, c). Similarly, pretreatment with SB203580 decreased the expression levels of IL-8 by 43.2 ± 5.6% and 66.1 ± 5.8% at the at mRNA and protein levels (Figure 4b, d). The involvement of p38 MAPK was further confirmed by the fact that HIV-1 Tat-mediated increase in phosphorylated p38 levels were decreased by pretreatment with SB203580 (Figure 4e).

**Figure 5:**
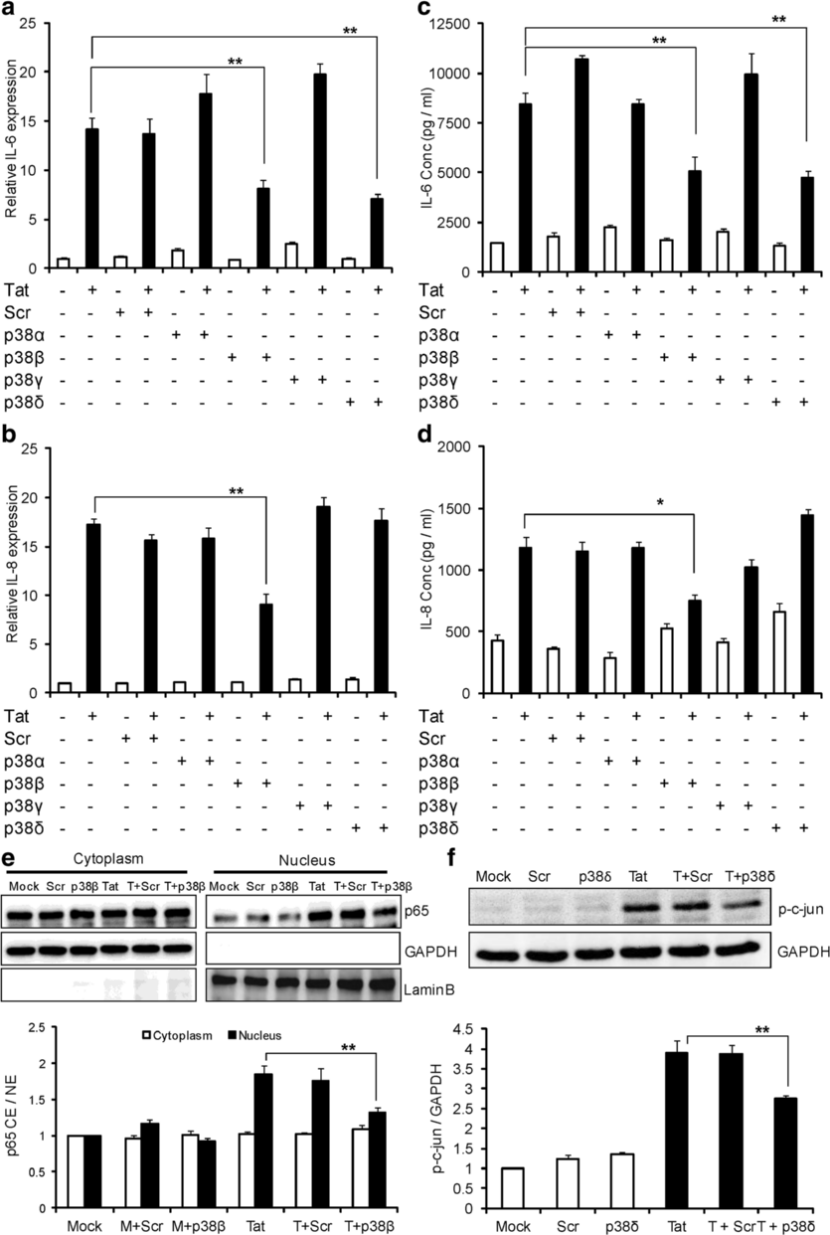
**Involvement of p38 mitogen activated-protein kinase (MAPK) in the induction of IL-6 and IL-8 by HIV-1 Tat. (a-f)** SVG astrocytes were transfected with siRNA (scrambled or p38α or p38β or p38γ or p38δ) for 48 hours. Then they are mock-transfected or transfected with plasmid encoding HIV-1 Tat. **(a, b)** The expression levels of IL-6 and IL-8 at mRNA were determined by real time RT-PCR at 6 hours post transfection. The values represented are normalized to their mock-transfected controls. **(c, d)** The protein concentrations of IL-6 and IL-8 were determined in the cell culture supernatants at 48 hours post transfection by multiplex cytokine assay. **(e)** Astrocytes were transfected with p38β siRNA for 48 hours prior to transfection with HIV-1 Tat plasmid. The translocation of p65 is measured 6 hours after transfection. Open bars and closed bars represent cytoplasm and nuclear fractions, respectively. **(f)** Astrocytes were transfected with p38δ siRNA for 48 hours, followed by transfection with HIV-1 Tat plasmid. The levels of p-c-jun were measured after 6 hours of transfection. A representative Western blot is shown in panels **(e)** and **(f)**. Each experiment was done at least in triplicate and each bar represents the ± SE of three individual experiments. Statistical analyses was performed by one-way ANOVA and ** denotes *P*-value of ≤ 0.01.

p38 MAPK exists in four different isoforms (α/β/γ/δ) of which SB203580 affects only p38α and p38β isoforms of p38 but not the other two isoforms (p38γ and p38δ). In order to validate the roles of p38α and p38β and also to determine the roles of the other two isoforms in the up-regulation of IL-6 and IL-8, we individually knocked down all the isoforms of p38 (α/β/γ/δ) using siRNA. The gene knock down was generally partial (>60%) except in the case of p38β where silencing was complete [29,30]. Knocking down p38β and p38δ isoforms decreased the expression of IL-6. Specifically, knock down of p38β decreased the expression of IL-6 by 42.6 ± 5.6% and 41.4 ± 4.9% at the levels of mRNA and protein, respectively (Figure 5a, c). Further, p38δ knock down decreased the expression of IL-6 by 49.7 ± 3.5% and 43.8 ± 4.3% at mRNA and protein levels respectively (Figure 5a, c). Individual knock down of only p38β decreased the expression level of IL-8 by 47.1 ± 6.4% and 36.2 ± 6.4% at the level of mRNA and protein respectively (Figure 5b, d). Of all the p38 isoforms, p38α and p38β are known to activate NF-κB. To see if p38 MAPK was leading to the activation of NF-κB, we pretreated the astrocytes with SB203580 and measured p65 translocation into the nucleus. Pretreatment with SB203580 decreased the translocation of p65 by 33.9 ± 4.6% compared to HIV-1 Tat-transfected astrocytes (Figure 4f). To specifically determine if p38β isoform led to the activation of NF-κB in our model system, we individually knocked down p38β isoform with siRNA and measured the translocation of p65 into the nucleus. Knocking down p38β isoform decreased p65 translocation by 28.5% ± 3.6% compared to HIV-1 Tat- transfected cells (Figure 5e).

### HIV-1 Tat-mediated induction of IL-6 and IL-8 involves AP-1 transcription factor activated by different upstream signaling molecules

**Figure 6:**
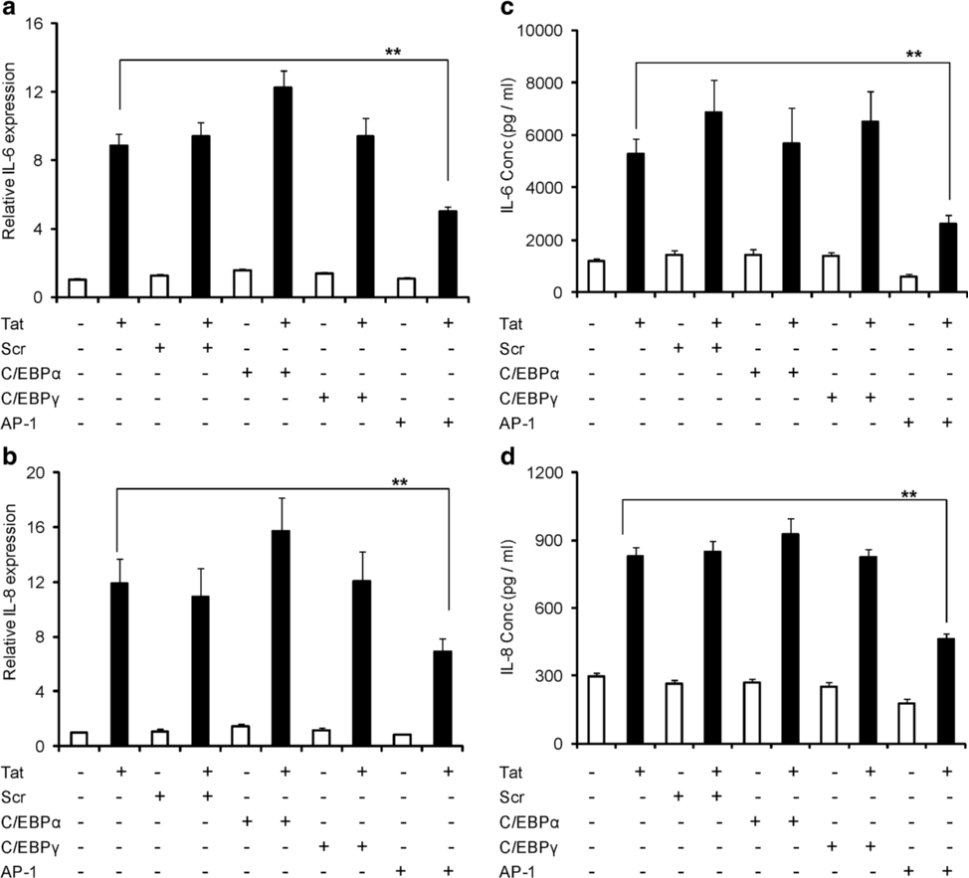
**HIV-1 Tat-mediated expression of IL-6 and IL-8 involves activator protein-1 (AP-1) transcription factor. (a-d)** Astrocytes were transfected with either scrambled or C/EBPα or C/EBPγ or AP-1 siRNA for a duration of 48 hours, followed by either mock transfection or transfection with HIV-1 Tat plasmid. **(a, b)** The expression levels of IL-6 and IL-8 at mRNA were determined by real time RT-PCR at 6 hours post transfection. The values represented are normalized to their mock-transfected controls. **(c, d)** Multiplex cytokine assay was employed to measure the protein concentrations of IL-6 and IL-8 in the cell culture supernatants at 48 hours post transfection. Each experiment was done at least in triplicate and each bar represents the ± SE of three individual experiments. Statistical analyses was performed by one-way ANOVA and ** denotes *P*-value of ≤ 0.01.

In view of our results that p38δ knock down decreased IL-6 expression; we wanted to determine the possible transcription factors that could be activated by p38δ. Activator protein-1 (AP-1) and CCAT enhancer binding proteins (C/CAT), specifically C/EBPα and C/EBPγ are known to be activated by p38δ. To ascertain the involvement of these transcription factors in the up-regulation of IL-6 and IL-8 by HIV-1 Tat, we individually knocked them down using specific siRNA. AP-1 (c-jun component) but not C/EBPα or C/EBPγ knock down by siRNA decreased the IL-6 expression by 43.2 ± 3.4% and 51.2 ± 6.3% at mRNA and protein level, respectively (Figure 6a, c). The role of AP-1 in IL-6 induction was further confirmed by time-dependent increase in expression of p-c-jun in both SVG astrocytes and primary astrocytes after exposure with HIV-1 Tat (Additional file 1: Figure S1A, B). To demonstrate that p38δ leads to the activation of AP-1, we knocked down p38δ and measured the levels of p-c-jun. Knocking down p38δ decreased the p-c-jun levels in the cells transfected with HIV-1 Tat by 30.8% ± 2.1% and scrambled siRNA did not have any effect on the p-c-jun levels (Figure 5f).

**Figure 7:**
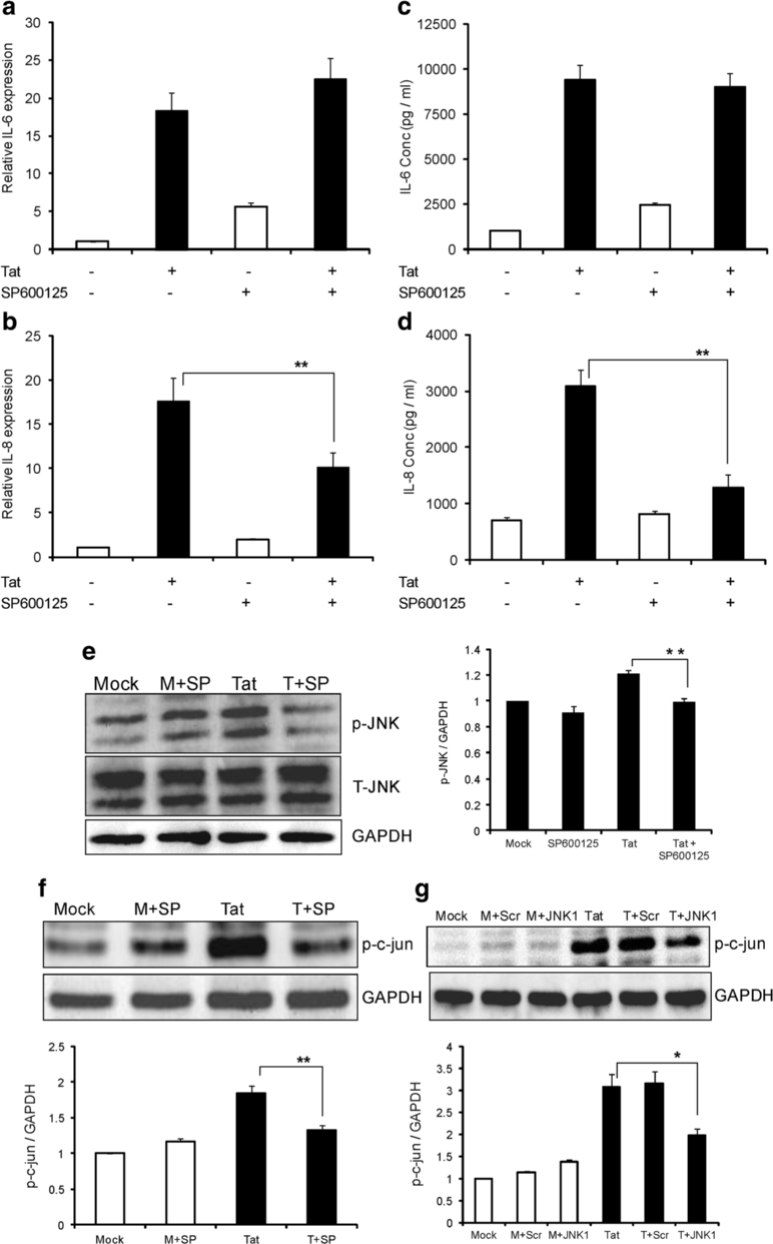
**Involvement of C-Jun N-terminal kinase/mitogen-activated protein kinase (JNK MAPK) in HIV-1 Tat-mediated expression of IL-8. (a-f)** SVG astrocytes were pretreated with JNK MAPK inhibitor (SP600125) for 1 hour prior to transfection. **(a, b)** The expression levels of IL-6 and IL-8 at mRNA level were determined at 6 hours post transfection by real time RT-PCR. The values represented are normalized to their mock- transfected controls. **(c, d)** IL-6 and IL-8 protein concentrations in the cell culture supernatants at 48 hours post transfection were determined by multiplex cytokine assay. **(e)** Astrocytes were either mock-transfected or transfected with HIV-1 Tat plasmid for duration of 6 hours and p-JNK and JNK were measured in whole cell extracts. **(f)** The levels of p-c-jun were measured in whole cell extracts 6 hours after the transfection. **(g)** Astrocytes were transfected with JNK1 siRNA for 48 hours, followed by transfection with HIV-1 Tat plasmid. The levels of p-c-jun were measured after 6 hours of transfection. A representative Western blot is shown in the panels **(e)**, **(f)** and **(g)**. Each experiment was done at least in triplicate and each bar represents the ± SE of three individual experiments. Statistical analyses was performed by one-way ANOVA and ** denotes *P*-value of ≤ 0.01.

Surprisingly, knocking down AP-1 transcription factor decreased the levels of IL-8 by 42.1 ± 8.4% and 44.3 ± 3.2% at the levels of mRNA and protein, respectively (Figure 6b, d). We next wanted to explore the other possible upstream signaling molecules that can be involved in the activation of AP-1. C-Jun N-terminal kinase (JNK) is another upstream signaling molecule that can activate AP-1. To determine the role of JNK in the up-regulation of IL-8 in astrocytes by HIV-1 Tat, we pretreated cells with a specific JNK inhibitor (SP600125) and measured the levels of IL-8 at 6 hours and 48 hours for mRNA and protein, respectively. Pretreatment with SP600125 decreased the levels of IL-8 by 42.4 ± 9.0% and 58.3 ± 7.0% at mRNA and protein levels, respectively (Figure 7b, d). We pretreated astrocytes with SP600125 and measured the phosphorylation of JNK to verify the activation of JNK MAPK by HIV-1 Tat. Pretreatment with SP600125 decreased the HIV-1 Tat-mediated increase in phosphorylated JNK (Figure 7e). Next, to determine whether JNK MAPK can lead to the activation of AP-1 transcription factor, we pretreated the astrocytes with SP600125 and measured the phosphorylation of c-jun. As shown in Figure 7f, densitometric analysis of Western blot shows that pretreatment with SP600125 decreased the levels of p-c-jun by 28.3 ± 3.3% compared to the HIV-1 Tat-transfected cells. To confirm the role of JNK MAPK in the activation of AP-1, we specifically knocked down JNK1 isoform using siRNA and measured the phosphorylated c-jun levels. Knock down of JNK1 decreased p-c-jun levels by 26.1 ± 5.2% compared to HIV-1 Tat-transfected cells (Figure 7g).

### Up-regulation of IL-6 and IL-8 by HIV-1 Tat involves PI3K/Akt pathway

**Figure 8:**
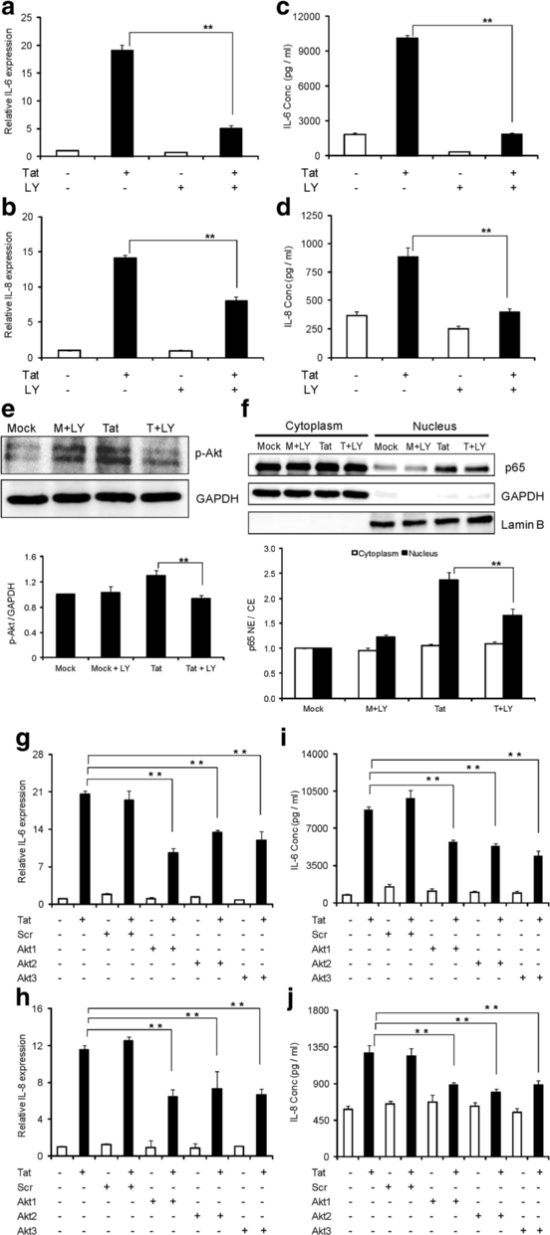
**HIV-1 Tat-mediated expression of IL-6 and IL-8 involves PI3K/Akt pathway. (a-f)** Astrocytes were pretreated with a specific PI3K inhibitor (LY294002) for 1 hour prior to transfection. **(a, b)** The expression levels of IL-6 and IL-8 at the level of mRNA were determined at 6 hours post transfection by real time RT-PCR. The values represented are normalized to their mock-transfected controls. **(c, d)** IL-6 and IL-8 protein concentrations in the cell culture supernatants at 48 hours post transfection were determined by multiplex cytokine assay. **(e)** Astrocytes were either mock-transfected or transfected with HIV-1 Tat plasmid for duration of 6 hours and p-Akt levels were measured in whole cell extracts. **(f)** Astrocytes were transfected for a duration of 6 hours and translocation of p65 was measured. Glyceraldehyde 3-phosphate dehydrogenase (GAPDH) and LaminB were used as internal loading controls for cytoplasmic and nuclear protein fractions, respectively. A representative Western blot is shown in figures **(e)** and **(f)**. **(g-j)** Astrocytes were transfected with either scrambled or Akt1 or Akt2 or Akt3 siRNA for a duration of 48 hours, followed by either mock transfection or transfection with HIV-1 Tat plasmid. **(g, h)** The levels of IL-6 and IL-8 at mRNA level were determined by real time RT-PCR at 6 hours post transfection. **(i, j)** The protein concentrations of IL-6 and IL-8 in cell culture supernatants at 48 hours post transfection were determined by multiplex cytokine assay. Each experiment was done at least in triplicate and each bar represents the ± SE of three individual experiments. Statistical analyses was performed by one-way ANOVA and ** denotes *P*-value of ≤ 0.01 and * denotes *P*-value of ≤ 0.05.

After determining the involvement of NF-κB and MAPK regulators in the up-regulation of IL-6 and IL-8, we wanted to explore the role of further upstream signaling molecules that can activate NF-κB. The phosphatidylinositol-4,5-bisphosphate 3-kinase (PI3K)/Akt pathway is a major upstream signaling mechanism that is involved in the regulation of cytokines by activation of NF-κB through IκB kinase (IKK)-mediated phosphorylation of IκBα [31]. To ascertain the role of PI3K/Akt in the up-regulation of IL-6 and IL-8 cytokines by HIV-1 Tat, we initially employed an inhibitor approach wherein the cells were pretreated with LY294002, a reversible PI3K inhibitor. LY294002 decreased HIV-1 Tat-mediated expression of IL-6 by 73.5 ± 2.6% and 81.3 ± 0.7% at the levels of mRNA and protein, respectively (Figure 8a, c). IL-8 was also decreased by 43.3 ± 3.5% and 55.1 ± 1.9% at mRNA and protein levels, respectively (Figure 8b, d). HIV-1 Tat-mediated increase in phosphorylated Akt levels were decreased by pretreatment with LY294002 (Figure 8e). Activation of NF-κB by PI3K was determined by measuring the translocation of p65 upon pretreatment with LY294002. Compared to HIV-1 Tat-transfected cells, pretreatment with LY294002 decreased the translocation of p65 by 29.9 ± 5.9% (Figure 8f). In order to confirm the results of a pharmacological antagonist, we employed the siRNA approach to individually knock down immediate downstream effector signaling molecules, including Akt1, Akt2 and Akt3. Individual knock down of all the isoforms decreased the expression levels of IL-6 and IL-8 by 35% and 30%, respectively at mRNA level (Figure 8g, h). Similar results were obtained at protein level, where the decrease for IL-6 and IL-8 was found to be 35% and 28%, respectively (Figure 8i, j).

## Discussion

Several mechanisms, including the dysregulation of the cytokine profile and infiltration of various inflammatory cells have been proposed for the development of HAND in patients infected with HIV-1 [32]. Elevated levels of pro-inflammatory cytokines including IL-6, IL-8, and IFN-γ have been found in the various regions of the brain in HIV infected people [33–35]. Particularly, elevated levels of IL-6 and IL-8 have been shown to play an important role in many inflammatory responses such as recruitment of leukocytes, accumulation of neutrophils and production of acute phase proteins [36,37]. Viral proteins, especially HIV-1 Tat and gp120, have been implicated in this phenomenon. Previous studies have shown the up-regulation of IL-6 and IL-8 by HIV-1 Tat in astrocytes [24,38]. However, very little is known about the molecular mechanisms behind the up-regulation of these cytokines. In the present study, we sought to dissect the molecular mechanisms behind the up-regulation of IL-6 and IL-8 cytokines by HIV-1 Tat in SVG astrocytes. The astrocytes were transfected with an expression plasmid encoding HIV-1 Tat and the expression levels of IL-6 and IL-8 were determined at various time points. The results showed a time-dependent increase in the expression of IL-6 and IL-8 at the level of mRNA and protein. These results are in agreement with the previous literature showing the over-expression of IL-6 and IL-8 induced by HIV-1 Tat [24,39]. However, the secretion of IL-6 was different compared to that shown in the previous studies [39]. This can be attributed to the difference in the type of cell line used (CRT-MG versus SVG astrocytes) and also different methods of HIV-1 Tat exposure (protein treatment versus transfection). However, the expression of IL-8 at protein level was consistent to that obtained by Kutch *et al*., where they demonstrated increased IL-8 protein expression with HIV-1 Tat protein treatment in primary astrocytes [24]. Our results with immunocytochemistry also confirm the elevated presence of IL-6 and IL-8 in HIV-1 Tat-transfected astrocytes. In our studies we transfected SVG astrocytes in most of the experiments whereas primary astrocytes were treated with recombinant protein. The transfection reflects the effect of ongoing infection whereas exogenous exposure reflects the effect of protein produced by same or other cells.

NF-κB is a major transcription factor involved in regulating the expression of many cytokines and chemokines. It binds to the promoter region of many genes, including IL-6 and IL-8 to regulate their expression [40]. Previous studies have shown increased binding and activation of NF-κB by HIV-1 Tat in various cells, including astrocytes and microglia [28,41]. A recent study by Fiume and co-workers has shown that HIV-1 Tat increased the binding of p65 DNA and also its transcriptional activity [42]. In agreement with these reports, we have also observed a time-dependent increase in the translocation of p65 into the nucleus by HIV-1 Tat in SVG astrocytes. Next, we demonstrated the involvement of NF-κB in the up-regulation of IL-6 and IL-8 by pretreating the astrocytes with BAY 11-7082, a specific inhibitor of IκB kinase 2 (IKK2). To confirm the data with the pharmacological inhibitor, we have employed the siRNA approach. NF-κB is comprised of different subunits of which p65 and p50 are important in regulating the expression of several cytokines/chemokines. The transcriptional activity is mainly attributed to the p65 subunit of NF-κB [43]. The 2 subunits were knocked down individually by using siRNA and expression of IL-6 and IL-8 were measured. Knock down of p65 but not p50 decreased the expression levels of both IL-6 and IL-8 in astrocytes by HIV-1 Tat. This suggests the possibility that p65 homodimers are more important in regulating the expression of IL-6 and IL-8 from astrocytes by HIV-1 Tat. This is in agreement with the findings by Georganas and co-workers showing that p65 homodimers but not p50 homodimers are important in regulating the expression of both IL-6 and IL-8 in rheumatoid arthritis fibroblast-like synoviocytes [44]. Surprisingly, knock down of p50 increased the expression levels of IL-8 at the mRNA and protein level. This can be attributed to the role of p50 homodimer as transcriptional repressor [45]. Therefore, it seems that HIV-1 Tat does not use p50 homodimer for up-regulating the expression of IL-8. However, further investigation needs to be done to determine the role of p50 homodimer on the expression of IL-8.

After determining the role of NF-κB, we sought to look at the role of upstream signaling molecules that can lead to up-regulation of IL-6 and IL-8 by HIV-1 Tat. MAPKs are important upstream signaling molecules that can result in the activation of many cytokines mediated through NF-κB. Particularly, p38 MAPK, belonging to the family of serine/threonine protein kinases has been shown to be involved in the up-regulation of many cytokines/chemokines [46]. The involvement of p38 MAPK in the up-regulation of IL-6 and IL-8 by HIV-1 Tat was determined by pretreating the astrocytes with SB203580, a specific inhibitor of p38 MAPK. p38 MAPK exists in four different isoforms (α/β/γ/δ), of which SB203580 inhibits the p38α and p38β isoforms [47]. Both these isoforms lead to the activation of NF-κB [48]. To verify the involvement of NF-κB activation (p38α and p38β isoforms) and the role of other two isoforms (p38γ and p38δ) in the up-regulation of IL-6 and IL-8 by HIV-1 Tat, we have individually knocked down all the isoforms using siRNA. Consistent with the results of SB203580, knock down of p38β but not p38α partially decreased the expression levels of IL-6 and IL-8. The decrease in the translocation of p65 into the nucleus with SB203580 pretreatment demonstrated the connection between p38 MAPK and NF-κB. Specifically, decrease in the translocation of p65 upon knock down of p38β shows that this isoform is more important for the activation of NF-κB by HIV-Tat. As knock down of p38δ partially reduced the expression levels of IL-6 and it does not lead to the activation of NF-κB, we wanted to determine the role of various other transcription factors that can be activated by it. p38δ activation is associated with increased activation of various transcription factors, including AP-1, C/EBPα and C/EBPγ [49]. Our results demonstrated that knock down of c-jun (important component of AP-1) but not C/EBPα and C/EBPγ has partially decreased the expression levels of IL-6. The Western blot showing a decrease in the phosphorylation of c-jun by HIV-1 Tat upon knock down of the p38δ isoform demonstrates that this isoform is important in HIV-1 Tat-mediated expression of IL-6.

As p38δ knock down did not alter the expression levels of IL-8 mediated by HIV-1 Tat, we were surprised to see the decreased expression levels of IL-8 by HIV-1 Tat upon knock down of AP-1. Then we tested the possibility of other upstream signaling molecules that can activate c-jun to induce the expression levels of IL-8 by HIV-1 Tat. Several previous studies have shown the role of JNK MAPK in the activation of c-jun [50]. JNK belongs to the family of MAPK and is involved in the activation of transcription factors in response to various stimuli. We demonstrated the role of JNK MAPK in the expression of IL-8 by HIV-1 Tat by pretreatment with SP600125, a specific inhibitor of JNK. It acts by competing with ATP to inhibit the phosphorylation of c-jun. Pretreatment of astrocytes with SP600125 and also siRNA against JNK has decreased the phosphorylation levels of c-jun by HIV-1 Tat. These results unequivocally demonstrate that JNK MAPK is important in the expression of IL-8 mediated by HIV-1 Tat.

PI3K/Akt is a major signaling molecule that can modulate the activation of NF-κB by promoting the phosphorylation of IκBα [51]. Several previous studies have indicated the role for HIV-1 and HIV-1 Tat in the activation of PI3K/Akt pathway in macrophages [52,53]. In our study, pretreatment of SVG astrocytes with the reversible PI3K inhibitor, LY294002, decreased HIV-1 Tat-mediated increase in the phosphorylation of PI3K. It also partially decreased HIV-1 Tat-mediated increase in IL-6 and IL-8. Akt or protein kinase B is a downstream signaling molecule of PI3K. It belongs to the family of serine/threonine protein kinases and is known to exist in three different isoforms (Akt1/PKBα, Akt2/PKBβ, Akt3/PKBγ). All three isoforms of Akt are present in the brain and differ in their phosphorylation sites [54]. In our study, individual knock down of all the isoforms by siRNA has decreased the expression levels of IL-6 and IL-8 mediated by HIV-1 Tat. These findings indicate the importance of all the isoforms in the expression of IL-6 and IL-8 mediated by HIV-1 Tat.

## Conclusions

**Figure 9:**
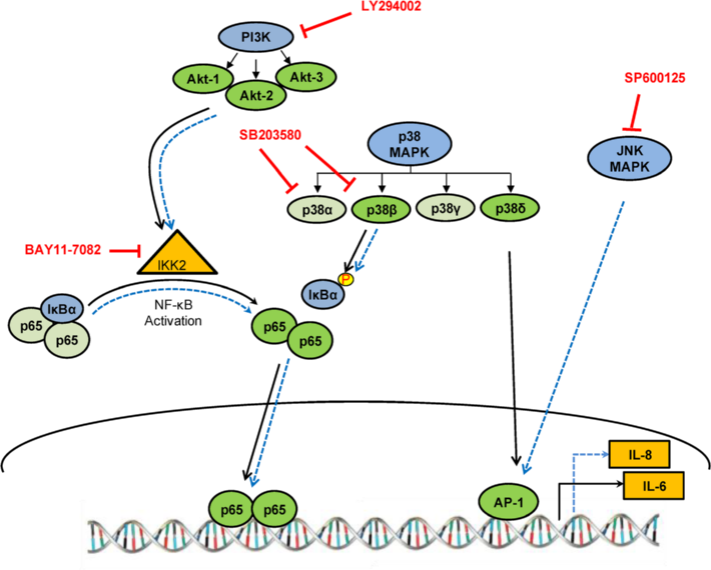
**Schematic of signaling pathways involved in HIV-1 Tat-mediated up-regulation of IL-6 and IL-8 in astrocytes.** The major signaling pathways involved in HIV-1 Tat-mediated up-regulation of IL-6 are PI3K/Akt and p38 MAPK which lead to the activation of NF-κB and AP-1 (solid line). Induction of IL-8 by HIV-1 Tat involves PI3K/Akt, p38 and C-Jun N-terminal kinase/mitogen activated-protein kinase (JNK MAPK) signaling pathways, leading to the activation of NF-κB and AP-1 transcription factors (broken line). The siRNAs used to target various isoforms are indicated by a green color whereas drug targets are indicated by a blue color. The involvement and absence of a particular isoform or a signaling molecule is indicated by a dark color and a pale color, respectively. Specific inhibitors used for targeting signaling molecules are indicated by a red color.

In conclusion, we have demonstrated that HIV-1 Tat induces the expression of IL-6 and IL-8 in astrocytes in a time-dependent manner at both mRNA and protein level. We demonstrated the involvement of various upstream signaling molecules, including PI3K/Akt, p38 MAPK and JNK MAPK by the use of pharmacological inhibitors and gene knock down using siRNA. We have similarly shown the activation of various transcription factors (NF-κB and AP-1) for HIV-1 Tat-mediated induction of IL-6 and IL-8 (Figure 9). As elevated levels of pro-inflammatory cytokines are implicated in the pathogenesis of HAND, blocking them presents a therapeutic intervention.

## Additional file

Additional file 1: Figure S1. HIV-1 Tat mediated up-regulation of p-c-jun in SVG astrocytes and primary astrocytes: (A) SVG astrocytes were either mock-transfected or transfected with HIV-1 Tat plasmid and p-c-jun protein levels were measured at 3, 6, 9 and 12 hours. Each experiment was done at least in triplicate and each bar represents the mean ± SE of three individual experiments. (B) Primary astrocytes were treated with 200 ng/mL Tat protein and p-c-jun protein levels were measured from 0 minutes to 60 minutes. The blot shown in Figure 3b was re-probed with p-c-jun antibody and the same GAPDH from Figure 3b is shown here. The bar graph represents the mean values obtained from two independent donors. Statistical analyses was performed by one-way ANOVA and ** denotes *P*-value of ≤ 0.01 and * denotes *P*-value of ≤ 0.05.

## Abbreviations

ANOVA: analysis of variance
AP-1: activator protein-1
BSA: bovine serum albumin
C/CAT: CCAT enhancer binding proteins
DAPI: 4′,6-diamidino-2-phenylindole
DMEM: Dulbecco’s modified Eagle’s medium
GAPDH: glyceraldehyde 3-phosphate dehydrogenase
GFAP: glial fibrillary acidic protein
HAD: HIV-associated dementia
HAND: HIV-associated neurocognitive disorders
HIV-1 Tat: HIV-1 trans activator of transcription
HRP: horseradish peroxidase
IKK2: inhibitor of IκB kinase 2
IKK: inhibitory kinase kinase
IL: interleukin
IFN: interferon
JNK: C-Jun N-terminal kinase
MCP-1: monocyte chemotactic protein 1
NF-κB: nuclear factor kappa B
p38 MAPK: p38 mitogen activated-protein kinase
PBST: phosphate-buffered saline with Tween 20
PCR: polymerase chain reaction
PD: Parkinson’s disease
PI3K: phophatidylinositol-4,5-biphosphate 3-kinase
PKB: protein kinase B
PVDF: polyvinylidene fluoride
RIPA: radioimmunoprecipitation assay
TNF: tumor necrosis factor

## References

[1] Forloni G, Mangiarotti F, Angeretti N, Lucca E, De Simoni MG (1997). Beta-amyloid fragment potentiates IL-6 and TNF-alpha secretion by LPS in astrocytes but not in microglia. Cytokine.

[2] Nagatsu T, Sawada M (2005). Inflammatory process in Parkinson’s disease: role for cytokines. Curr Pharm Des.

[3] Maimone D, Guazzi GC, Annunziata P (1997). IL-6 detection in multiple sclerosis brain. J Neurol Sci.

[4] Kaul M, Garden GA, Lipton SA (2001). Pathways to neuronal injury and apoptosis in HIV-associated dementia. Nature.

[5] Yuan L, Qiao L, Wei F, Yin J, Liu L, Ji Y, Smith D, Li N, Chen D (2013). Cytokines in CSF correlate with HIV-associated neurocognitive disorders in the post-HAART era in China. J Neurovirol.

[6] Gonzalez-Scarano F, Martin-Garcia J (2005). The neuropathogenesis of AIDS. Nat Rev Immunol.

[7] Shah A, Verma AS, Patel KH, Noel R, Rivera-Amill V, Silverstein PS, Chaudhary S, Bhat HK, Stamatatos L, Singh DP, Buch S, Kumar A (2011). HIV-1 gp120 induces expression of IL-6 through a nuclear factor-kappa B-dependent mechanism: suppression by gp120 specific small interfering RNA. PLoS One.

[8] Yeung MC, Pulliam L, Lau AS (1995). The HIV envelope protein gp120 is toxic to human brain-cell cultures through the induction of interleukin-6 and tumor necrosis factor-alpha. AIDS.

[9] Toborek M, Lee YW, Pu H, Malecki A, Flora G, Garrido R, Hennig B, Bauer HC, Nath A (2003). HIV-Tat protein induces oxidative and inflammatory pathways in brain endothelium. J Neurochem.

[10] Nath A, Conant K, Chen P, Scott C, Major EO (1999). Transient exposure to HIV-1 Tat protein results in cytokine production in macrophages and astrocytes. A hit and run phenomenon. J Biol Chem.

[11] Cann AJ, Rosenblatt JD, Wachsman W, Shah NP, Chen IS (1985). Identification of the gene responsible for human T-cell leukaemia virus transcriptional regulation. Nature.

[12] Haughey NJ, Holden CP, Nath A, Geiger JD (1999). Involvement of inositol 1,4,5-trisphosphate-regulated stores of intracellular calcium in calcium dysregulation and neuron cell death caused by HIV-1 protein tat. J Neurochem.

[13] Midde NM, Gomez AM, Zhu J (2012). HIV-1 Tat protein decreases dopamine transporter cell surface expression and vesicular monoamine transporter-2 function in rat striatal synaptosomes. J Neuroimmune Pharmacol.

[14] El-Hage N, Podhaizer EM, Sturgill J, Hauser KF (2011). Toll-like receptor expression and activation in astroglia: differential regulation by HIV-1 Tat, gp120, and morphine. Immunol Invest.

[15] D’Aversa TG, Yu KO, Berman JW (2004). Expression of chemokines by human fetal microglia after treatment with the human immunodeficiency virus type 1 protein Tat. J Neurovirol.

[16] Andras IE, Pu H, Tian J, Deli MA, Nath A, Hennig B, Toborek M (2005). Signaling mechanisms of HIV-1 Tat-induced alterations of claudin-5 expression in brain endothelial cells. J Cereb Blood Flow Metab.

[17] Minagar A, Shapshak P, Fujimura R, Ownby R, Heyes M, Eisdorfer C (2002). The role of macrophage/microglia and astrocytes in the pathogenesis of three neurologic disorders: HIV-associated dementia, Alzheimer disease, and multiple sclerosis. J Neurol Sci.

[18] Thompson KA, McArthur JC, Wesselingh SL (2001). Correlation between neurological progression and astrocyte apoptosis in HIV-associated dementia. Ann Neurol.

[19] Navarrete M, Perea G, Fernandez de Sevilla D, Gomez-Gonzalo M, Nunez A, Martin ED, Araque A (2012). Astrocytes mediate in vivo cholinergic-induced synaptic plasticity. PLoS Biol.

[20] Halassa MM, Haydon PG (2010). Integrated brain circuits: astrocytic networks modulate neuronal activity and behavior. Annu Rev Physiol.

[21] Gorry PR, Ong C, Thorpe J, Bannwarth S, Thompson KA, Gatignol A, Vesselingh SL, Purcell DF (2003). Astrocyte infection by HIV-1: mechanisms of restricted virus replication, and role in the pathogenesis of HIV-1-associated dementia. Curr HIV Res.

[22] Churchill MJ, Wesselingh SL, Cowley D, Pardo CA, McArthur JC, Brew BJ, Gorry PR (2009). Extensive astrocyte infection is prominent in human immunodeficiency virus-associated dementia. Ann Neurol.

[23] Dou H, Morehead J, Bradley J, Gorantla S, Ellison B, Kingsley J, Smith LM, Chao W, Bentsman G, Volsky DJ, Gendelman H (2006). Neuropathologic and neuroinflammatory activities of HIV-1-infected human astrocytes in murine brain. Glia.

[24] Kutsch O, Oh J, Nath A, Benveniste EN (2000). Induction of the chemokines interleukin-8 and IP-10 by human immunodeficiency virus type 1 tat in astrocytes. J Virol.

[25] Chen P, Mayne M, Power C, Nath A (1997). The Tat protein of HIV-1 induces tumor necrosis factor-alpha production. Implications for HIV-1-associated neurological diseases. J Biol Chem.

[26] Nookala AR, Shah A, Noel RJ, Kumar A (2013). HIV-1 Tat-mediated induction of CCL5 in astrocytes involves NF-kappaB, AP-1, C/EBPalpha and C/EBPgamma transcription factors and JAK, PI3K/Akt and p38 MAPK signaling pathways. PLoS One.

[27] Shah A, Kumar A (2010). HIV-1 gp120-mediated increases in IL-8 production in astrocytes are mediated through the NF-kappaB pathway and can be silenced by gp120-specific siRNA. J Neuroinflammation.

[28] Nicolini A, Ajmone-Cat MA, Bernardo A, Levi G, Minghetti L (2001). Human immunodeficiency virus type-1 Tat protein induces nuclear factor (NF)-kappaB activation and oxidative stress in microglial cultures by independent mechanisms. J Neurochem.

[29] Gangwani MR, Noel RJ, Shah A, Rivera-Amill V, Kumar A (2013). Human immunodeficiency virus type 1 viral protein R (Vpr) induces CCL5 expression in astrocytes via PI3K and MAPK signaling pathways. J Neuroinflammation.

[30] Liu X, Shah A, Gangwani MR, Silverstein PS, Fu M, Kumar A (2014). HIV-1 Nef induces CCL5 production in astrocytes through p38-MAPK and PI3K/Akt pathway and utilizes NF-kB, CEBP and AP-1 transcription factors. Sci Rep.

[31] Kane LP, Shapiro VS, Stokoe D, Weiss A (1999). Induction of NF-kappaB by the Akt/PKB kinase. Curr Biol.

[32] Rappaport J, Joseph J, Croul S, Alexander G, Del Valle L, Amini S, Khalili K (1999). Molecular pathway involved in HIV-1-induced CNS pathology: role of viral regulatory protein, Tat. J Leukoc Biol.

[33] Mamik MK, Ghorpade A (2012). Src homology-2 domain-containing protein tyrosine phosphatase (SHP) 2 and p38 regulate the expression of chemokine CXCL8 in human astrocytes. PLoS One.

[34] Perrella O, Guerriero M, Izzo E, Soscia M, Carrieri PB (1992). Interleukin-6 and granulocyte macrophage-CSF in the cerebrospinal fluid from HIV infected subjects with involvement of the central nervous system. Arq Neuropsiquiatr.

[35] Griffin DE, McArthur JC, Cornblath DR (1991). Neopterin and interferon-gamma in serum and cerebrospinal fluid of patients with HIV-associated neurologic disease. Neurology.

[36] Gabay C (2006). Interleukin-6 and chronic inflammation. Arthritis Res Ther.

[37] Baggiolini M, Clark-Lewis I (1992). Interleukin-8, a chemotactic and inflammatory cytokine. FEBS Lett.

[38] El-Hage N, Gurwell JA, Singh IN, Knapp PE, Nath A, Hauser KF (2005). Synergistic increases in intracellular Ca2+, and the release of MCP-1, RANTES, and IL-6 by astrocytes treated with opiates and HIV-1 Tat. Glia.

[39] Ju SM, Song HY, Lee JA, Lee SJ, Choi SY, Park J (2009). Extracellular HIV-1 Tat up-regulates expression of matrix metalloproteinase-9 via a MAPK-NF-kappaB dependent pathway in human astrocytes. Exp Mol Med.

[40] Matsusaka T, Fujikawa K, Nishio Y, Mukaida N, Matsushima K, Kishimoto T, Akira S (1993). Transcription factors NF-IL6 and NF-kappa B synergistically activate transcription of the inflammatory cytokines, interleukin 6 and interleukin 8. Proc Natl Acad Sci U S A.

[41] Conant K, Ma M, Nath A, Major EO (1996). Extracellular human immunodeficiency virus type 1 Tat protein is associated with an increase in both NF-kappa B binding and protein kinase C activity in primary human astrocytes. J Virol.

[42] Fiume G, Vecchio E, De Laurentiis A, Trimboli F, Palmieri C, Pisano A, Falcone C, Pontoriero M, Rossi A, Scialdone A, Fasanella Masci F, Scala G, Quinto I (2012). Human immunodeficiency virus-1 Tat activates NF-kappaB via physical interaction with IkappaB-alpha and p65. Nucleic Acids Res.

[43] Vallabhapurapu S, Karin M (2009). Regulation and function of NF-kappaB transcription factors in the immune system. Annu Rev Immunol.

[44] Georganas C, Liu H, Perlman H, Hoffmann A, Thimmapaya B, Pope RM (2000). Regulation of IL-6 and IL-8 expression in rheumatoid arthritis synovial fibroblasts: the dominant role for NF-kappa B but not C/EBP beta or c-Jun. J Immunol.

[45] Plaksin D, Baeuerle PA, Eisenbach L (1993). KBF1 (p50 NF-kappa B homodimer) acts as a repressor of H-2Kb gene expression in metastatic tumor cells. J Exp Med.

[46] Lee JC, Laydon JT, McDonnell PC, Gallagher TF, Kumar S, Green D, McNulty D, Blumenthal MJ, Heys JR, Landvatter SW, Strickler JS, Mclauglin MM, Siemens IR, Fisher SM, Livi GP, White JR, Adams JL, Young PR (1994). A protein kinase involved in the regulation of inflammatory cytokine biosynthesis. Nature.

[47] Lee JC, Kassis S, Kumar S, Badger A, Adams JL (1999). p38 mitogen-activated protein kinase inhibitors–mechanisms and therapeutic potentials. Pharmacol Ther.

[48] Kumar V, Behera R, Lohite K, Karnik S, Kundu GC (2010). p38 kinase is crucial for osteopontin-induced furin expression that supports cervical cancer progression. Cancer Res.

[49] Efimova T, Broome AM, Eckert RL (2003). A regulatory role for p38 delta MAPK in keratinocyte differentiation. Evidence for p38 delta-ERK1/2 complex formation. J Biol Chem.

[50] Papachristou DJ, Batistatou A, Sykiotis GP, Varakis I, Papavassiliou AG (2003). Activation of the JNK-AP-1 signal transduction pathway is associated with pathogenesis and progression of human osteosarcomas. Bone.

[51] Heck S, Lezoualc’h F, Engert S, Behl C (1999). Insulin-like growth factor-1-mediated neuroprotection against oxidative stress is associated with activation of nuclear factor kappaB. J Biol Chem.

[52] Lucas A, Kim Y, Rivera-Pabon O, Chae S, Kim DH, Kim B (2010). Targeting the PI3K/Akt cell survival pathway to induce cell death of HIV-1 infected macrophages with alkylphospholipid compounds. PLoS One.

[53] Chugh P, Bradel-Tretheway B, Monteiro-Filho CM, Planelles V, Maggirwar SB, Dewhurst S, Kim B (2008). Akt inhibitors as an HIV-1 infected macrophage-specific anti-viral therapy. Retrovirology.

[54] Easton RM, Cho H, Roovers K, Shineman DW, Mizrahi M, Forman MS, Lee VM, Szabolcs M, de Jong R, Oltersdorf T, Ludwig T, Efstratiadis A, Birnbaum MJ (2005). Role for Akt3/protein kinase Bgamma in attainment of normal brain size. Mol Cell Biol.

