# Supplementary figures and images for: Molecular mechanisms involved in HIV-1 Tat-mediated induction of IL-6 and IL-8 in astrocytes
Source: J Neuroinflammation. 2014 Dec 24;11:214. doi: 10.1186/s12974-014-0214-3 (PMC4302610; doi:10.1186/s12974-014-0214-3)

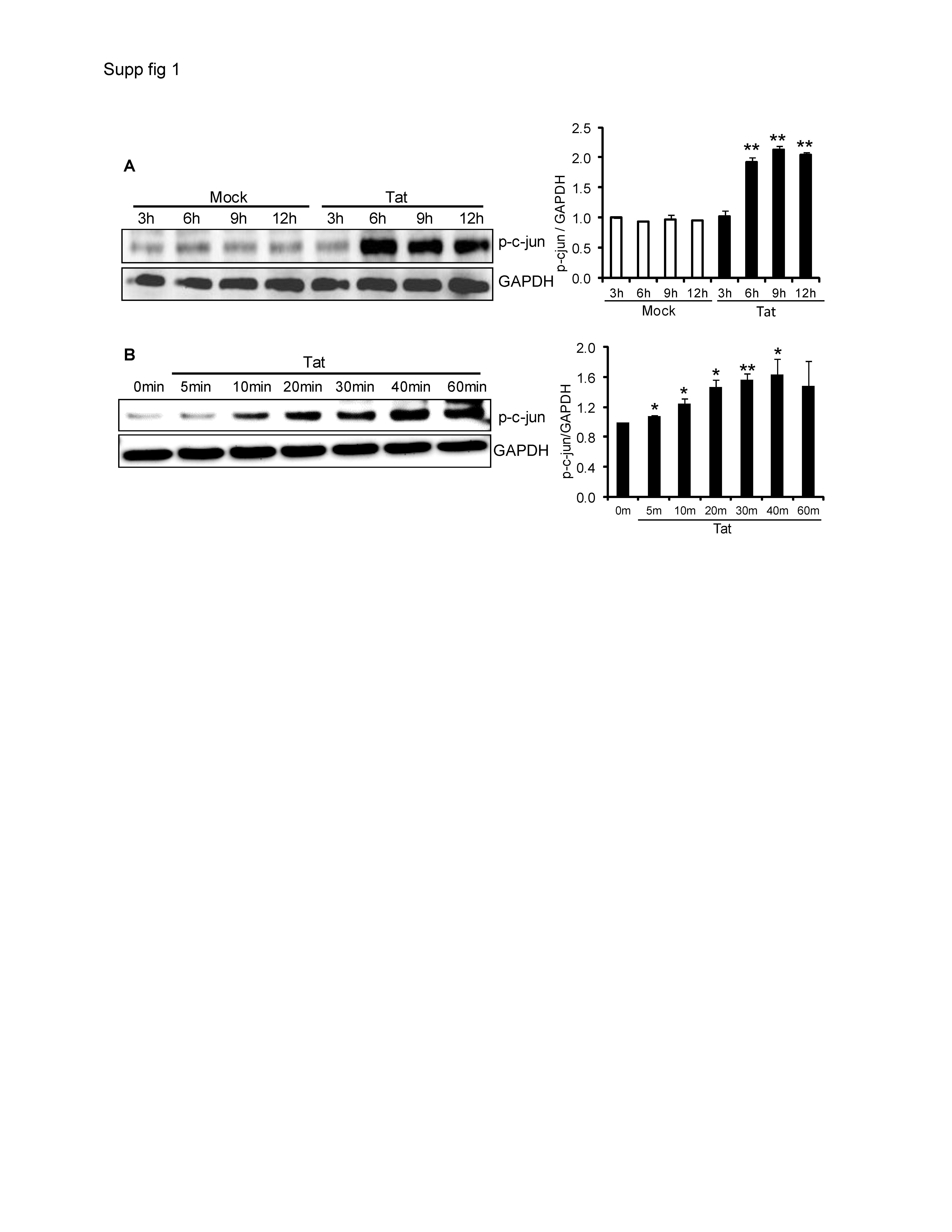

Supplement: Additional file 1: Figure S1. — HIV-1 Tat mediated up-regulation of p-c-jun in SVG astrocytes and primary astrocytes: (A) SVG astrocytes were either mock-transfected or transfected with HIV-1 Tat plasmid and p-c-jun protein levels were measured at 3, 6, 9 and 12 hours. Each experiment was done at least in triplicate and each bar represents the mean ± SE of three individual experiments. (B) Primary astrocytes were treated with 200 ng/mL Tat protein and p-c-jun protein levels were measured from 0 minutes to 60 minutes. The blot shown in Figure 3b was re-probed with p-c-jun antibody and the same GAPDH from Figure 3b is shown here. The bar graph represents the mean values obtained from two independent donors. Statistical analyses was performed by one-way ANOVA and ** denotes P-value of ≤ 0.01 and * denotes P-value of ≤ 0.05. [file 12974_2014_214_MOESM1_ESM.tiff]
